# RNA Interference of the Ecdysone Receptor Genes *EcR* and *USP* in Grain Aphid (*Sitobion avenae* F.) Affects Its Survival and Fecundity upon Feeding on Wheat Plants

**DOI:** 10.3390/ijms17122098

**Published:** 2016-12-14

**Authors:** Ting Yan, Hongmei Chen, Yongwei Sun, Xiudao Yu, Lanqin Xia

**Affiliations:** Institute of Crop Sciences, Chinese Academy of Agricultural Sciences (CAAS), Beijing 100081, China; nmgyanting@sina.com (T.Y.); chenchang1934@163.com (H.C.); nuliba_hehe@163.com (Y.S.); yuxiudao@163.com (X.Y.)

**Keywords:** grain aphid (*Sitobion avenae* F.), ecdysone receptor (EcR), ultraspiracle protein (USP), RNA interference (RNAi)

## Abstract

RNA interference (RNAi) has been widely used in functional genomics of insects and received intensive attention in the development of RNAi-based plants for insect control. Ecdysone receptor (EcR) and ultraspiracle protein (USP) play important roles in molting, metamorphosis, and reproduction of insects. *EcR* and *USP* orthologs and their function in grain aphid (*Sitobion avenae* F.) have not been documented yet. Here, RT-PCR, qRT-PCR, dsRNA feeding assay and aphid bioassay were employed to isolate *EcR* and *USP* orthologs in grain aphid, investigate their expression patterns, and evaluate the effect of RNAi on aphid survival and fecundity, and its persistence. The results indicated that *SaEcR* and *SaUSP* exhibited similar expression profiles at different developmental stages. Oral administration of dsRNAs of *SaEcR* and *dsSaUSP* significantly decreased the survival of aphids due to the down-regulation of these two genes, respectively. The silencing effect was persistent and transgenerational, as demonstrated by the reduced survival and fecundity due to knock-down of *SaEcR* and *SaUSP* in both the surviving aphids and their offspring, even after switching to aphid-susceptible wheat plants. Taken together, our results demonstrate that *SaEcR* and *SaUSP* are essential genes in aphid growth and development, and could be used as RNAi targets for wheat aphid control.

## 1. Introduction

Aphids are major agricultural pests in crop plants due to their direct feeding and vectoring various destructive plant viruses [1,2]. Each year, the worldwide crop losses due to aphid infestation are estimated at hundreds of millions of dollars [3,4,5]. The major aphid species infesting wheat (*Triticum aestivum* L) include grain aphid (*Sitobion avenae* F.), Russian wheat aphid (*Diuraphis noxia*), greenbug (*Schizaphis graminum* Rondani), bird-cherry oat aphid (*Rhopalosiphum padi* Linnaeus), and rose-grain aphid (*Metopolophium dirhodum* Walker). Of these, the grain aphid is the most dominant and destructive, and represents a major pest of wheat in China, Europe, and North America [6,7,8,9]. In the absence of genetic plant resistance, insecticide treatments remain the main way for aphid control. However, excessive dependence on insecticides is undesirable because of the development of insecticide resistance, the potential negative effects on non-target organisms, and environmental pollution [8]. Outbreaks of aphids causing substantial losses are reported regularly. For example, in 2015–2016 crop seasons, approximately 70% of the total growth area of wheat suffered aphid infestations in China (available online: http://www.natesc.moa.gov.cn). Therefore, breeders are still struggling to find an alternative strategy for aphid control in wheat [8,9,10].

Expression of double strand RNA (dsRNA) designed against insect target genes in transgenic plants has been shown to give protection against pests through RNA interference (RNAi), opening the way for a new generation of insect-resistant crops [8,11]. RNAi refers to dsRNA mediated gene silencing [12]. Since its discovery in *Caenorhabditis elegans* [12], it has been widely used in insect genetic research [13] and recently received intensive attention in the development of RNAi-based plants for insect control (for review, please see [8,10]). However, not all the target genes are sensitive to dsRNA treatment. The efficacy of RNAi-mediated knockdown appears to depend on the identity and nature of the target gene [14]. For example, of 290 dsRNAs tested in western corn rootworm, only 14 genes showed significant mortality and/or stunting at low dsRNA concentrations of 0.52 ng/cm^2^ [15]. Among a set of 66 unigenes in grain aphid selected for a dsRNA artificial diet assay, only four effective RNAi targets were identified [16]. Furthermore, 5490 unigenes were found to be differentially expressed upon feeding in the alimentary canals of grain aphid. Sixteen significant up- and down-regulated genes were selected for dsRNA treatment, and among them, only five of them were effective RNAi targets [9]. It was proposed that the RNAi target gene should be a crucial gene for insect development or survival, thus causing lethal effect, growth inhibition, or reduced fecundity once knocked-down. Additionally, the essential genes involved in herbivory, metamorphosis, or key metabolic processes might be potential RNAi targets [10].

Ecdysteroids play an important role in biological processes such as development, molting, metamorphosis, and reproduction in insects [17,18,19,20,21]. Ecdysone receptor (EcR) and ultraspiracle protein (USP) are two essential molting hormone receptors involved in growth and development of insects [22]. EcR binds to ecdysone and functions through heterodimerization with USP. Ecdysteroids promote binding of the EcR/USP heterodimer to a specific DNA ecdysone response element (EcRE) that is located in the promoter region of a series of ecdysteroid-responsive genes and thereafter triggers the expression of a cascade of genes involved in regulating some key developmental events in insects [20,23,24]. Since the discovery of *EcR* and *USP* genes in *Drosophila melanogaster* in the early 1990s [24,25,26], the *EcR* and *USP* orthologs from around 20 insects which belong to Diptera [27], Lepidoptera [28], Homoptera [29], Coleoptera [30], and Orthoptera [31], respectively, have been isolated. Both EcR and USP have common structural characteristics, these are: a variable N-terminal region (A/B domain); a central, well-conserved DNA-binding domain (DBD, also termed C domain); a non-conserved hinge (D domain); and a carboxy-terminus, moderately conserved ligand-binding domain (LBD, E domain), except that EcR has an additional F domain. Whereas C domain functions in dimerization and DNA binding, E domain has an important role in dimerization and ligand binding [22]. *EcR* and *USP* are critical for larval molting and pupal metamorphosis in red flour beetle, *Tribolium castaneum*. RNAi analysis showed that the knocking-down of these two genes led to lethal effect [32]. Expression of dsRNA derived from the *EcR* coding regions can effectively and specifically interfere with the functions of this gene in *Drosophila*, leading to defects in larval molting and metamorphosis in insects, thus causing them to fail to pupariate or prepupae. These observed developmental defects were caused by the disruption of the genetic cascades that control the onset of metamorphosis [33]. Furthermore, when *H. armigera* larvae were fed with leaves of transgenic tobacco plants expressing *EcR* dsRNA, its *EcR* mRNA level was dramatically decreased and resulted in molting defects and larval lethality [34]. Although *EcR* and *USP* have been isolated and characterized in different insect orders, the orthologs in grain aphid and their functions have not been documented yet.

In this study, orthologs of *EcR* and *USP* genes, *SaEcR* and *SaUSP*, were isolated from grain aphid. The expression profiles of these two genes at different developmental stages were characterized, and the effects of dsRNAs on the expression of *SaEcR* and *SaUSP*, the mortality and fecundity of aphids, and the persistent and transgenerational effect of RNAi were investigated in order to understand the roles of *SaEcR* and *SaUSP* in aphid growth and development, and provide information for application of these two genes as potential RNAi targets for aphid control.

## 2. Results

### 2.1. Isolation and Characterization of EcR and USP Genes from Grain Aphid

The encoding sequences of *EcR* and *USP* in grain aphid were isolated based on the contigs obtained from our previous transcriptome profiling of grain aphid [16] and their orthologs in pea aphid (AP-EcR, gi|641662353 and AP-USP, gi|239735515). The full-lengths of *EcR* and *USP* homologs in grain aphid were 1620 and 1305 bp, encoding a 540 and a 435 amino acids protein, and designated as *SaEcR* and *SaUSP*, respectively. Sequence alignment indicated that *SaEcR* and *SaUSP* shared 98.40% and 94.71%, and 99.44% and 93.09% identities to its counterparts in pea aphid (*A. pisum*) at nucleotide acid and amino acid levels, respectively (Figure S1). As indicated in Figure S1, only several nucleoid differences in *EcR* were observed between grain aphid and pea aphid, whereas big differences were observed around an 81 bp region at 5′ terminal of *USP*. Further analysis of the deduced amino acid sequences of SaEcR and SaUSP using the SMART (Simple Modular Architecture Research Tool) program (available online: http://smart.embl-heidelberg.de) revealed that they both had the same typical conserved domains as their counterparts in other insects, such as transactivation domain (A/B domain), DNA binding domain (C domain), D domain, and ligand binding domain (E), except for SaEcR which had an additional F domain.

**Figure 1:**
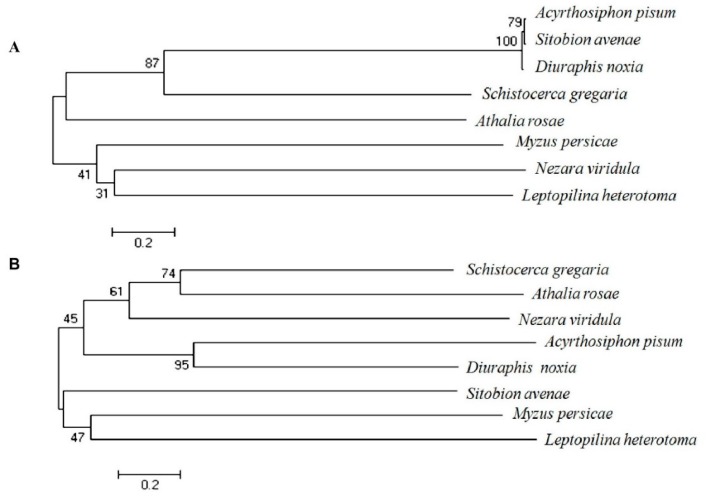
Phylogenetic trees of the ecdysone receptor (EcR) and ultraspiracle protein (USP) in grain aphid and other insect species. Insect phylogenetic trees showed the phylogenetic relationships of the species addressed in this study. (**A**) Evolutionary relationships between the EcR of grain aphid and those of other insects; (**B**) Evolutionary relationships between the USP of grain aphid and those of other insects. The joint enrooted tree was generated using MEGA 6.0 by the minimum evolution method. Bootstrap values were indicated at each branch.

To analyze the evolutionary relationships among the insect species in which EcR and USP orthologs showed high homology with that of grain aphid at amino acid level, phylogenetic trees of EcR and USP in several aphid species and other insects were constructed through MEGA 6.0 software (available online: www.megasoftware.net) by using the full-length amino acid sequences as a matrix. For sequences of EcR, those insect species included Russian wheat aphid (*Diuraphis noxia*) (XP_015375738.1), peach aphid (*Myzus persicae*) (ABN11289.1), pea aphid (*Acyrthosiphon pisum*) (NP_001152832.1), *Athalia rosae* (XP_012253055.1), *Schistocerca gregaria* (ALO17613.1), *Nezara viridula* (ADQ43370.1), and *Leptopilina heterotoma* (AAO18154.1). For sequences of USP, those insect species included Russian wheat aphid (*Diuraphis noxia*) (XP_015369584.1), peach aphid (*Myzus persicae*) (ABN11290.1), pea aphid (*Acyrthosiphon pisum*) (NP_001155140.1), *Nezara viridula* (ADQ43369.1), *Schistocerca gregaria* (ALO17614.1), *Leptopilina heterotoma* (AAO18153.1), and *Athalia rosae* (XP_012262809.1). Phylogenetic analysis indicated SaEcR had closer evolutionary relationships with its orthologs in pea aphid and peach aphid, whereas SaUSP only showed a closer relationship with that of pea aphid (Figure 1).

Furthermore, using BLAST on the NCBI database (available online: www.ncbi.nlm.nih.gov/blast) demonstrated that *SaEcR* had 68%, 69%, and 78% sequence homology to its orthologs in aphid enemies *Harmonia axyridis* (GenBank: AB506665.1), *Henosepilachna vigintioctopunctata*) (GenBank: AB506669.1), and *Agelena silvatica* (GenBank: GQ281317.1), respectively, whereas *SaUSP* showed 76%, 66%, and 72% homology with its counterparts from aphid enemies *Harmonia axyridis* (GenBank: AB506667.1), *Henosepilachna vigintioctopunctata* (GenBank: AB506671.1), and *Agelena silvatica* (GenBank: HM102368.1), respectively. However, no continuous three 21-nt matches were observed between *SaEcR* and *SaUSP* and their counterparts from these insect species at the nucleotide acid level (data not shown), therefore suggesting that the dsRNAs derived from the coding sequences of these two genes would be potentially safe for non-target organisms.

**Figure 2:**
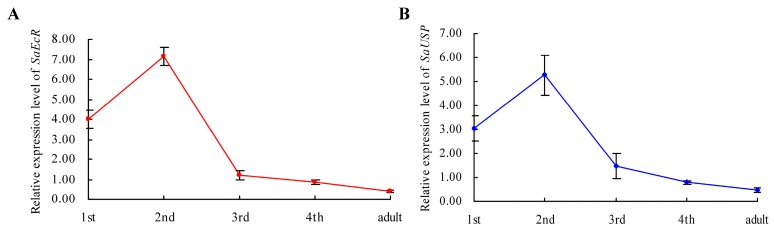
The expression profiles of *SaEcR* (**A**); and *SaUSP* (**B**) at different developmental stages in grain aphid. *SaEcR* and *SaUSP* transcripts accumulated at various levels throughout the aphid development. The expression of both *SaEcR* and *SaUSP* were simultaneously up-regulated and peaked in the second instar nymphs, then decreased gradually. Bars represent mean values ± SEM (standard error of the mean) of three independent biological replicates, each with a pool of 10 individual aphids.

Moreover, the expression profiles of *SaEcR* and *SaUSP* at different developmental stages, namely the first, second, third, and fourth instar nymphs and adults of grain aphid, were detected by using quantitative real-time PCR (qRT-PCR) with *actin* gene and ribosomal protein L27 (*L27*) gene as internal controls. Results showed that *SaEcR* and *SaUSP* transcripts accumulated at various levels throughout different developmental stages (Figure 2). The expression patterns of both *SaEcR* and *SaUSP* were similar, up-regulated, and peaked in the second instar nymphs, then decreased gradually from the third instar stage, while the lowest levels were observed in the adults. As indicated in Figure 2, the expression levels of *SaEcR* and *SaUSP* were 21.2 and 11.8 fold higher in the second instar nymphs compared to these of the adults, respectively.

### 2.2. The Effects of dsRNAs of SaEcR and SaUSP on Aphid Development and Mortality

**Figure 3:**
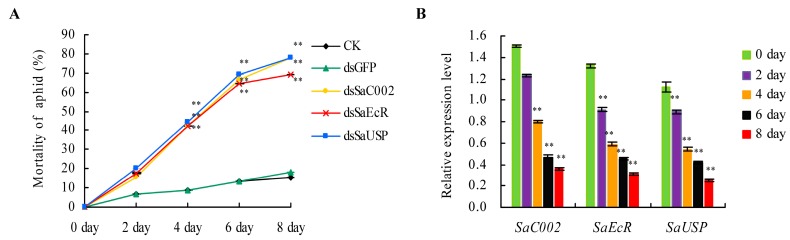
Mortalities and the relative expression levels of *SaEcR* and *SaUSP* of grain aphid at different time points upon feeding on artificial diet added with dsRNAs. (**A**) The mortality of the second instars of grain aphid fed on artificial diet added with dsRNA. CK, represents the blank artificial diet control without dsRNA. dsGFP represents the artificial diet with added dsRNA of *GFP* as a negative control; dsC002 represents the artificial diet with added dsRNA of *C002* as a positive control. The dsRNAs were added to the artificial diet at a concentration of 7.5 ng/μL. The survival of the nymphs was monitored over an eight day period. Values and error bars reflect the mean and SEM of six independent biological replicates, each with a pool of 30 individual aphids (Student’s *t*-test, *n* = 6, ** *p* < 0.01); (**B**) The relative expression levels of *SaC002*, *SaEcR*, and *SaUSP* genes upon feeding on artificial diet containing dsRNA at different time points. Quantitative real-time PCRs (qRT-PCRs) were performed using the total RNA from the survived instars at different time points after feeding on artificial diet containing the respective dsRNAs. The relative expression levels of target genes were monitored at indicated time points over an eight day period. The expression levels of both genes were knocked-down significantly after dsRNA feeding at day 4 and thereafter. Values and error bars represent the mean and SEM of three independent biological replicates, each with a pool of eight surviving individual aphids (Student’s *t*-test, *n* = 3, ** *p* < 0.01).

To investigate for potential application of these two genes in dsRNA-mediated RNAi for aphid control, dsRNAs of *SaEcR* and *SaUSP* were synthesized and incorporated into an artificial diet. *C002* is a salivary gland-specific gene, encoding an important effector involved in aphid interaction with host plants. Disruption of this gene through plant-mediated RNAi improved aphid resistance of transgenic tobacco plants [35,36]. DsRNA of *SaC002* (dsSaC002) from grain aphid, which was identified and named as *SaC002* in our previous study, was used a positive control, whereas *GFP* dsRNA (dsGFP) was used as a negative control [9]. We used the 7.5 ng/μL concentration of dsRNA to investigate the mortality of the second instar grain aphids feeding on artificial diet added with dsRNA of *SaEcR* or *SaUSP* (dsSaEcR or dsSaUSP). As shown in Figure 3A, when dsRNA was added to the artificial diet, four days later, the mortalities of aphids fed with dsSaC002, dsSaEcR, and dsSaUSP were around 50% after correction, respectively. At day 8, increased mortality rates of 68%–80% were observed for dsSaC002, dsSaEcR, and dsSaUSP treatments, which were significantly higher than the control treatments with dsGFP or without dsRNA at all (Student’s *t*-test, *n* = 6, ** *p* < 0.01). Given the fact that compared with blank the artificial diet without any dsRNA added (control), the dsGFP in the artificial diet had no effect on the mortality of aphids, the lethality mentioned above was thus caused by a sequence-specific effect of the dsRNA rather than by the physical or chemical characteristics of dsRNAs per se [9].

To investigate the relationship between the aphid mortality and the expression level of the respective target gene, the relative expression levels of *SaEcR* and *SaUSP* were investigated in aphid larvae at different time points after feeding upon the artificial diet added with the respective dsRNA by qRT-PCRs using the aphid *actin* and *L27* genes as internal controls. The relative expression levels of *SaEcR* and *SaUSP* were monitored at indicated time points over an eight day period. As indicated in Figure 3B, *SaEcR* and *SaUSP* transcripts decreased by 28.7% and 20.0%, respectively, after dsSaEcR and dsSaUSP feeding at day 2. The expression of *SaEcR* and *SaUSP* were knocked-down significantly after dsRNA feeding at day 4, and declined thereafter to as high as 82.6% and 85.0% after feeding at day 8 (Student’s *t*-test, *n* = 3, ** *p* < 0.01), respectively, whereas the administration of dsRNA did not affect the expression levels of household genes *actin* and *L27* (data not shown). These results indicated that the mortality and developmental stunting caused by dsRNA related feeding was due to the down-regulation of the respective target genes, and the reduced *SaEcR* and *SaUSP* expression levels correlated well with a decline in growth, reproduction, and survival rates. Taken together with the results of the mortality analyses, it is clear that *SaEcR* and *SaUSP* are good candidate targets for aphid control through the implementation of a plant-mediated RNAi strategy for agricultural practices.

### 2.3. Persistent Silencing and Transgenerational Effects of RNAi in Grain Aphid after Switching onto Aphid-Susceptible Wheat Plants

**Figure 4:**
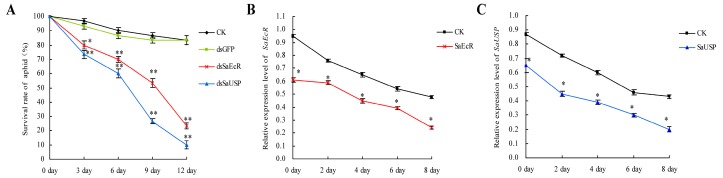
Persistent silencing effects of RNAi in grain aphid. (**A**) Effects of *SaEcR* and *SaUSP* silencing on the survival rate of grain aphid after transferring onto wheat plants. The survival rate of aphids after dsRNA feeding continued to decrease significantly even after transferring to wheat plants (Student’s *t*-test, *n* = 3, * *p* < 0.05, ** *p* < 0.01). Bars represent mean values ± SEM of three independent biological replicates, each with a pool of 30 individual aphids; (**B**) Expression profile of *SaEcR* after transferring to wheat plants (Student’s *t*-test, *n* = 3, * *p* < 0.05). Bars represent mean values ± SEM three independent biological replicates, each with a pool of eight surviving individual aphids; (**C**) Expression profile of *SaUSP* after transferring to wheat plants (Student’s *t*-test, *n* = 3, * *p* < 0.05). Bars represent mean values ± SEM of three independent biological replicates, each with a pool of eight surviving individual aphids.

To investigate the persistence of silencing effects of dsSaEcR and dsSaUSP, second instar nymphs of grain aphid were fed on artificial diet with dsSaEcR and dsSaUSP for three days to interfere with the target genes, and then the surviving aphids were transferred onto the aphid-susceptible wheat (*Triticum aestivum* L. cv. Beijing 837) plants by using aphids derived from feeding only pure artificial diet and dsGFP as a blank control (CK) and a negative control. After three days, the survival rates of *S. avenaes* fed on the wheat plants decreased to around 70% (Figure 4A). The survival rates kept declining along with feeding on wheat plants. At day 12, the survival rates of aphid in the dsSaEcR group and dsSaUSP group dropped to 23.3% and 10.0%, respectively, rates that were significantly lower than that of the control group (Student’s *t*-test, *n* = 3, ** *p* < 0.01), whereas one obvious difference in survival rate was observed between dsGFP treatment and CK. To assess the expression levels of the target genes in the surviving aphids on wheat plants, qRT-PCR was performed by using RNA extracted from surviving aphids every two days for up to eight days. As indicated in Figure 4B,C, the expression levels of *SaEcR* and *SaUSP* in surviving aphids on wheat plants after dsRNA treatments continued decreasing and were significantly lower than control (Student’s *t*-test, *n* = 3, * *p* < 0.05). The decreased expression levels of the target genes were closely correlated to the mortality levels of aphids, indicating the persistence of the RNAi effect.

The persistence of RNAi effects on aphid fitness were investigated by feeding synchronous second instar aphids for three days on artificial diet with added dsRNA, then transferring the surviving aphids to aphid-susceptible wheat plants and rearing them for five to eight days to allow them to grow to adults and reproduce. Various fitness parameters such as survival, adult longevity (day), fecundity (day), daily fecundity, and total production were evaluated in an aphid bioassay. As indicated in Table 1, the adult longevity (day), daily fecundity, fecundity (day), and total production of the dsSaEcR and dsSaUSP treatments were significantly decreased, two to six folds lower, than those of the control group (Student’s *t*-test, *n* = 3, ** *p* < 0.01), whereas no difference was observed in those of the dsGFP treatment compared to CK.

**Figure 5:**
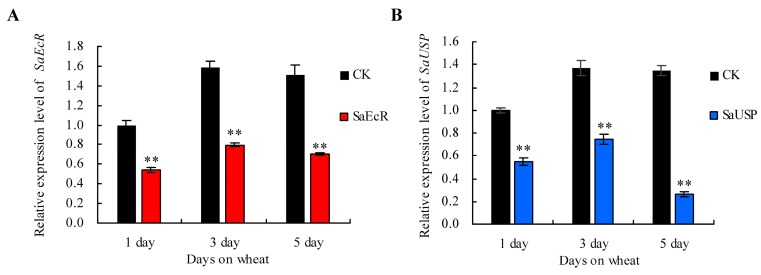
Transgenerational effect of RNAi in the offspring of grain aphids exposed to dsRNAs treatment. The relative expression levels of *SaEcR* (**A**); and *SaUSP* (**B**) in neonate nymphs of the aphids exposed to dsRNAs treatment were significantly lower than those fed on CK control (Student’s *t*-test, *n* = 3, ** *p* < 0.01). Bars represent mean values ± SEM of three independent biological replicates, each with a pool of eight individual aphids.

The potential transgenerational RNAi effects of *SaEcR* and *SaUSP* were investigated by using the newborn nymphs produced in a parallel experiment. The newborn nymphs (1 day) were transferred individually inside clip cages on fresh wheat plants and collected at different time points such as 1, 3, and 5 day. We then monitored *SaEcR*/*SaUSP* expression in the successive next generation of aphids fed solely on wheat plants by qRT-PCR. As indicated in Figure 5, the expression levels of *SaEcR* and *SaUSP* were significantly reduced in the offspring of aphids exposed to dsRNA treatment compared with the control, indicating that the dsRNA-mediated RNAi effect not only persisted in the parental generation, but also led to the target gene knock-down in the successive offspring.

## 3. Discussion

We demonstrated here that the silencing of *SaEcR* and *SaUSP*, two functional receptors for the insect molting hormone ecdysone, significantly decreased the survival and fecundity of grain aphids due to the down-regulation of these two genes. In *T. Castaneum*, *Drosophila*, and *Helicoverpa armigera*, the silencing of *EcR* and/or *USP* resulted in molting defects and mortality of the insect juveniles [32,34], indicating that *EcR* and *USP* indeed play an important role in development and reproduction across different insect orders. However, our result contrasts with the absences of RNAi responses in pea aphid when dsRNAs targeting *EcR* and *USP* genes were delivered through either oral feeding or injection [37]. Differences in RNAi efficacy have been widely observed between experiments and laboratories, even in different populations or strains of the same species in different labs [8,10,14,38]. In this case, it is possible that the target region for dsRNA design, the life stage of the aphids for dsRNA oral administration, and the duration of dsRNA feeding might account for the differences. In this study, dsEcR and dsUSP were designed to target the E domain of EcR and USP, which is supposed to function in ligand binding and plays important roles in the heterodimerization of EcR and USP (Figure S1). Furthermore, we used second instar nymphs to perform the dsRNA feeding assay rather than neonate nymphs. The growth rate of grain aphid is relatively higher at the second and third instar stages than others, and thus higher corresponding nutrient requirements means a greater amount of dsRNA absorption. Moreover, we monitored the survival and development of aphids and the target gene expression at different time points over an eight day period, although decreased survival of aphids and reduced expression of target genes were observed even after three days of dsRNA feeding (Figure 2).

Remarkably, we found that this dsRNA-mediated silencing effect through oral administration was persistent and transgenerational, as demonstrated by the reduced survival and expression levels of *SaEcR* and *SaUSP* in both the surviving aphids and their offspring even after switching to aphid-susceptible wheat plants (Figure 3, Figure 4 and Figure 5). Silencing in insects could be achieved by continuous feeding with either single-stranded siRNA or dsRNA [39], and vertically transmitted from parental RNAi to their progeny [40]. Similar phenomena were documented in several studies [39,41,42]. For example, RNAi-based *C002* transcript knockdown dramatically reduces the life span and leads to the premature deaths of pea aphids (*Acyrthosiphon pisum*) on fava bean leaves. After transferring the aphids injected with siRNA of *C002* to fava bean plants, several foraging or feeding parameters can be affected in a statistically significant way by the knock-down. Additionally, the aphid’s ability to identify a suitable location for initiating probing is significantly reduced because it takes *C002*-knockdown aphids six times longer than the control insects to identify such a site and begin probing [42]. *MpC002* and *MpPIntO2* (*Mp2*) are effector genes modulating aphid-plant interactions in peach aphid. Maximal reduction of gene expression of around 70% was achieved at between four and eight days of exposure of the peach aphids to *MpC002* and *MpPIntO2* dsRNA-producing transgenic *Arabidopsis thaliana*. Target genes were also down-regulated in nymphs born from mothers exposed to dsRNA-producing transgenic plants, and the RNAi effect lasted 12 to 14 days in these nymphs [39]. Our results are also consistent with the observed RNAi effect in barley in which host-induced gene silencing targeting *shp*, a structural sheath protein encoding gene, had a prolonged impact and caused strong transgenerational effects on feeding, development, and survival of grain aphid [41]. However, the mechanism underlying this long-lasting and transgenerational RNAi effect in insects remains unclear [40,41].

The identification of suitable RNAi targets is a prerequisite for aphid control through a plant-mediated RNAi strategy. The off-target silencing effect on non-target organism poses a potential risk of cross-species silencing and is a major biosafety concern in the application of a plant-mediated RNAi strategy for agricultural practices because some functional domains of certain genes are highly conserved across different organisms [10]. The specificity of RNAi requires a precise design of the region for dsRNA synthesis [43]. The observation that a shared sequence length of ≥21 nt was required for efficacy against the target gene and all active orthologs contained at least three 21-nt matches [44], provides a fundamental basis for screening potential RNAi targets in insects. In this study, although *SaEcR* and *SaUSP* had higher sequence homology to its orthologs in some aphid enemies, no continuous three 21-nt matches were observed between *SaEcR* and *SaUSP* and their counterparts from these non-target insect species at the nucleotide acid level, suggesting that the dsRNAs derived from the coding sequences of these two genes would be potentially safe for non-target organisms. However, it would still be valuable to perform laboratory dsRNA feeding experiments on likely non-target insects, especially the aphid enemies, to circumvent any potential non-target effects [10].

## 4. Materials and Methods

### 4.1. Plant and Insects

Wheat: 15–20 seeds of wheat (*Triticum aestivum* L. cv. Beijing 837, a susceptible-wheat variety to aphid infestation) were planted in pots (10 cm diameter) that were kept in a control room at 22 °C, with 40%–60% relative humidity, and a 16-h photoperiod. Plants at the two-leaf stage in each pot were enclosed in Perspex tubes which were sealed with porous plastic sheeting and were used for further experiments.

Aphids: Grain aphids (*Sitobion avenae* F.) were reared on two-leaf wheat seedlings in a control room. To obtain synchronized insects, apterous adult grain aphids derived from a single clonal lineage reared as a continuous culture on wheat seedlings were placed in cages (one aphid per cage) for 24 h to produce nymphs. Twenty neonate nymphs produced in a 24 h period were transferred into fresh wheat seedlings in each pot. Five days later, the second instar nymphs were selected and subjected to the artificial diet feeding experiment. Twelve days later, the offspring of these aphids at different development stages were collected from wheat seedlings with a brush and immediately frozen in liquid nitrogen and stored at −80 °C before RNA extraction for expression pattern analysis.

### 4.2. Isolation and Characterization of SaEcR and SaUSP Genes from Grain Aphid

Total RNA was extracted using Transzol UP (TransGen Biotech, Beijing, China) according to the manufacturer’s instructions. The extracted total RNA was examined by electrophoresis, quantified using UV spectrophotometer, and reverse transcribed into first strand cDNA using FastQuant RT Kit (Tiangen, Beijing, China). The synthesized first strand cDNA was used for subsequent gene amplification. The primer sets SaEcR-F/R, SaUSP-F/R and USPP-F/R used for cloning of *SaEcR* and *SaUSP* genes were designed using primer design software Primer 5.0 (Premier Biosoft, Palo Alto, CA, USA) based on the contigs generated from our previous transcriptome profiling of grain aphid [16] and the coding sequences of *EcR* and *USP* orthologs from pea aphid (*A. pisum*) (Table S1). The amplified DNA fragments were sequenced by HuaDa Gene (HuaDa Gene, Beijing, China).

DNA sequence data were analyzed by BLAST on the NCBI database (available online: http://www.ncbi.nlm.nih.gov/blast/) and the motifs and domains were identified using the SMART (Simple Modular Architecture Research Tool) program (available online: http://smart.embl.de/). The alignments of the coding sequence and deduced protein sequences between aphid species were performed using DNAMAN software (Lynnon Biosoft, San Ramon, CA, USA). The conserved motif and the full amino acid sequences of proteins were used for multiple sequence alignments and phylogenetic analysis by using ClustalW in MEGA 6.0 [45] and MEGA 6.0 [46] with the tree with a higher bootstrap value presented along with each set of sequences.

To examine the expression profiles of *SaEcR* and *SaUSP* at different developmental stages, namely in the first, second, third, and fourth instar nymphs and adults of grain aphid, 10 aphids at different developmental stages were collected and detected using quantitative real-time PCR (qRT-PCR) following the protocol described below with at least three technical replicates, respectively.

### 4.3. RNA Extraction and qRT-PCR Analyses

RNA was extracted from aphids using the Qiagen RNA Extraction kit (Qiagen, Beijing, China) according to manufacturer’s instructions. The sequences of primer sets, qSaUSP-F/R, qSaEcR-F/R qactin-F/R, and qL27-F/R were as listed in Table S1. Freshly extracted mRNA from aphids was converted into cDNA using Superscript II reverse transcriptase (Invitrogen, Beijing, China) according to manufacturer’s instruction. Quantitative real-time PCR (qRT-PCR) assay was performed using the SYBRH Premix Ex Taq^TM^ II (TaKaRa, Beijing, China) in an ABI 7300 Real Time PCR system. The expression levels of *actin* and *L27* were used to normalize the *C*t value obtained for each gene. All qRT-PCR experiments were repeated in triplicate. Then the mean value generated from the reference genes was used to calculate the relative gene expression of the respective target gene using the 2^−^^ΔΔ^*^C^*^t^ method, as previously described by Livak and Schmittgen [47].

### 4.4. dsRNA Design and Synthesis 

Before dsRNA synthesis, the dsRNA targeting region of *EcR* and *USP* of grain aphid were used to BLAST against the non-target organisms (available online: http://www.ncbi.nlm.nih.gov/blast/), including wheat (taxid: 4565), human (taxid: 9606) and the major natural enemies of aphids, such as Chrysopa (taxid: 76806), Asaphes vulgaris (taxid: 338020), Coccinellidae (taxid: 7080), Reduviidae (taxid: 27479), flower flies (taxid: 34680), Araneae (taxid: 6893), and Mantodea (taxid: 7504), to find if there were active homologous sequences that may cause off-target effect.

PCR products amplified from the dsRNA targeting regions of *EcR* and *USP* were purified using TIANgel Mini Purification Kit (Tiangen, Beijing, China), respectively, according to manufacturer’s instructions. T_7_ promotor sequences were tailed to each end of the DNA template by PCR amplifications. dsRNAs were synthesized using the MEGAscript RNAi kit (Ambion, Huntingdon, UK) according to the manufacturer’s instructions. Double-stranded RNA targeting *SaC002* (dsC002) was synthesized and used as positive control in the dsRNA artificial feeding experiments, and double-stranded GFP (dsGFP) was generated using pPigbacA3EYFP as a template and was used as a negative control in the dsRNA feeding experiments, as performed by Zhang et al. [9]. The lengths of dsRNAs synthesized ranged from 100 to 500 bp by using the primer sets T_7_SaEcR-F/R, T_7_SaUSP-F/R, T_7_SaC002-F/R, and T_7_GFP-F/R, respectively (Table S1). After the template DNA and single-stranded RNA were removed from the transcription reaction by DNase I and RNaseA treatments, the dsRNA was purified using MEGAclear^TM^ columns (Ambion, Huntingdon, UK) and eluted in 100 μL nuclease free water. The concentrations of dsRNAs were measured using Biophotometer (Eppendorf, Germany).

### 4.5. Artificial Diet Bioassay

Liquid artificial diet was prepared as described by Whyard et al. [48], and dsRNA artificial diet bioassay was performed according to Zhang et al. [9]. After one day of pre-feeding on blank artificial diet at 22–24 °C, 40%–60% relative humidity, and a photoperiod of 16:8 (Light:Dark), 30 nymphs (second instar) were transferred to a new vial which contained artificial diet added with dsRNAs with a fine paintbrush. The aphids fed with dsRNA of *GFP* gene, which has no homologous sequences in aphid, were used as a negative control, whereas the aphids fed with only diet were used as blank control. In addition, the aphids fed with dsSaC002, which was 307 bp in length and was proven to have lethal effect on aphid growth in grain aphid in our previous study [9], were used as a positive control. The bioassay vials were placed with the opening with gauze upside and incubated under the same conditions for pre-feeding. For each treatment, the number of surviving aphids were counted every two days over an eight day period. The data were then analyzed based on six replicates to investigate the effects of the dsRNA treatments on the development and mortality of aphids compared to those of the dsGFP and blank control. To assess the extent of RNAi, RNA was extracted from pools of eight dsRNA-treated and surviving aphids at different time points of dsRNA artificial feeding over an eight day period for further qRT-PCR analyses following the protocol as described above with at least three technical replicates.

### 4.6. Evaluation of the Persistence and Transgenerational Effects of RNAi after Switching onto Aphid-Susceptible Wheat Plants

The second instar nymphs were fed with artificial diet with added dsGFP, dsSaEcR, and dsSaUSP, and blank artificial diet, respectively, in a glass vial for three days. For each treatment, in total, 180 aphids in nine glass vials (20 aphids in each glass vial) were used with three glass vials treated as one replicate. Then 30 surviving aphids derived from each replicate were transferred onto wheat plants. The aphids were placed inside the clip cages separately with one aphid per cage. The survival of aphids was investigated every three days over a 12 day period. The relative expression levels of the *SaEcR* and *SaUSP* in a pool of eight surviving aphids were examined using qRT-PCR every two days in an eight day period with three replicates.

The persistence of RNAi effects on aphid fitness were investigated by feeding synchronous second instar aphids for three days on artificial diet with added dsRNA, then transferring the surviving aphids to aphid-susceptible wheat plants, placing them inside the clip cages individually, and rearing them for five to eight days to allow them to grow to adults and reproduce. The molting, fecundity, life span, and survival of aphids were recorded daily until the death of the adult. Adult longevity (day), fecundity (day), daily fecundity, and total production were then calculated. Each treatment was repeated in triplicate. For transgenerational effect analysis, five to eight days later after switching onto wheat plants, the neonate nymphs produced by adult aphids in a 24 h period were transferred to fresh wheat plants. Then the relative expression levels of *SaEcR* and *SaUSP* in a pool of eight offspring nymphs were investigated using qRT-PCR at one, three, and five days after being transferred onto wheat plants. Each treatment was repeated in triplicate. 

### 4.7. Statistical Analyses

The data of the dsRNA artificial diet assay were analyzed using one-way ANOVA to investigate the effects of the dsRNA treatment on the mortality of aphids compared to the blank control. The data of different aphid fitness parameters such as survival, mortality, fecundity, and total production in an aphid bioassay were analyzed by using SAS9.3 software (SAS Institute Inc., Cary, NC, USA). The differences between or among groups were examined using a student’s *t*-test. Significance (*p*-value) was evaluated at the 1% or 5% level for all comparisons. For each treatment, the standard error of the mean (SEM) was calculated based on at least three biological replicates.

## 5. Conclusions

RNAi of *SaEcR* and *SaUSP* through oral dsRNA administration significantly decreased the survival of grain aphids. Reduced *SaEcR* and *SaUSP* expression levels correlated well with a developmental delay and a decline in reproduction and survival rates. The RNAi effect persisted across the life stages of aphids from neonate nymph to adults, and transmitted from the parental lines to their offspring even after switching to aphid-susceptible wheat plants. Our results not only define that *SaEcR* and *SaUSP* are essential genes in aphid growth and reproduction in grain aphid, but also demonstrate the feasibility of RNAi-based strategy for aphid control in an agricultural practice.

## Figures and Tables

**Table 1:** The fitness parameters of grain aphids on wheat plants after dsRNA feeding.

Treatments	Adult Longevity (Day)	Fecundity Period (Day)	Daily Fecundity	Total Production
CK	10.30 ± 0.23	8.43 ± 0.15	1.50 ± 0.04	12.63 ± 0.53
dsGFP	10.00 ± 0.21	8.30 ± 0.23	1.41 ± 0.03	11.62 ± 0.16
dsSaEcR	4.73 ± 0.15 **	3.10 ± 0.23 **	0.33 ± 0.05 **	1.97 ± 0.19 **
dsSaUSP	3.83 ± 0.17 **	2.63 ± 0.05 **	0.34 ± 0.05 **	2.17 ± 0.09 **

Values shown in the table are means and standard error of mean. CK is blank control and dsGFP is negative control (Student’s *t*-test, *n* = 3, ** *p* < 0.01).

## Supplementary Materials

Supplementary materials can be found at www.mdpi.com/1422-0067/17/12/2098/s1.
