# Supplementary material for: RNA Interference of the Ecdysone Receptor Genes EcR and USP in Grain Aphid (Sitobion avenae F.) Affects Its Survival and Fecundity upon Feeding on Wheat Plants
Source: Int J Mol Sci. 2016 Dec 14;17(12):2098. doi: 10.3390/ijms17122098 (PMC5187898; doi:10.3390/ijms17122098)
Supplement: Supplementary file 1 [file ijms-17-02098-s001.pdf]

Supplementary Materials: RNA Interference of the Ecdysone Receptor Genes *EcR* and *USP* in Grain Aphid (*Sitobion avenae* F.) Affects Its Survival and Fecundity upon Feeding on Wheat Plants

Ting Yan, Hongmei Chen, Yongwei Sun, Xiudao Yu and Lanqin Xia

Table S1. Primer sets used in this study.

Use of Primers	Sequences of Primers (5′ → 3′)		Annealing Tempreture (°C)	Amplicon Length (bp)
cDNA cloning	SaEcR-F	CCACCGACTTGTCTATTTCCAGG	55 °C	429 bp
	SaEcR-R	GCATATTCTGCGTTATCCACCTTC		
	SaUSP-F	AGAGCTGGTTGGAATGAGTTG	52 °C	371 bp
	SaUSP-R	AATGAAGGAAGCCGAAGAAGT		
	SaC002-F	CCGACAAATTCAAAGAGACGAAGAC	55 °C	267 bp
	SaC002-R	AGAAACTTCCAAACTTATTCACGGC		
	GFP-F	CGGGAACTACAAGACACG	50 °C	320 bp
	GFP-R	CTTTGGAAAGGGCAGATT		
	USPP-F	TGGTTGGAATGAGTTGATG	56 °C	493 bp
	USPP-R	CATGTAGCTACTTGAACGTCATG		
dsRNA synthesis	T_7SaEcR-F	TAATACGACTCACTATAGGGCCACCGACTTGTCTATTTCCAGG	55 °C	469 bp
	T_7SaEcR-R	TAATACGACTCACTATAGGGGCATATTCTGCGTTATCCACCTTC		
	T_7SaUSP-F	TAATACGACTCACTATAGGGAGAGCTGGTTGGAATGAGTTG	55 °C	411 bp
	T_7SaUSP-R	TAATACGACTCACTATAGGGAATGAAGGAAGCCGAAGAAGT		
	T_7SaC002-F	TAATACGACTCACTATAGGCGACAAATTCAAAGAGACGAAGAC	55 °C	307 bp
	T_7SaC002-R	TAATACGACTCACTATAGGAGAAACTTCCAAACTTATTCACGGC		
	T_7GFP-F	TAATACGACTCACTATAGGGCGGGAACTACAAGACACG	50 °C	360 bp
	T_7GFP-R	TAATACGACTCACTATAGGGCTTTGGAAAGGGCAGATT		
qRT-PCR	qactin-F	CGGTTCAAAAACCCAAACCAG	57 °C	231 bp
	qactin-R	TGGTGATGATTCCCGTGTTC		
	L27-F	CCGAAAAGCTGTCATAATGAAGAC	57 °C	246 bp
	L27-R	GGTGAAACCTTGTCTACTGTTACATCTTG		
	qSaEcR-F	TGATGTTCAGGGTAGCAAGG	57 °C	137 bp
	qSaEcR-R	CCGACTGAATGACAGTTGGT		
	qSaUSP-F	ATGGGTATGGATAGAACAG	57 °C	198 bp
	qSaUSP-R	TAATGAAGGAAGCCGAAG		
	qSaC002-F	CCGACAAATTCAAAGAGACGAAGAC	57 °C	267 bp
	qSaC002-R	AGAAACTTCCAAACTTATTCACGGC		

T_7 RNA polymerase promoter is underline.

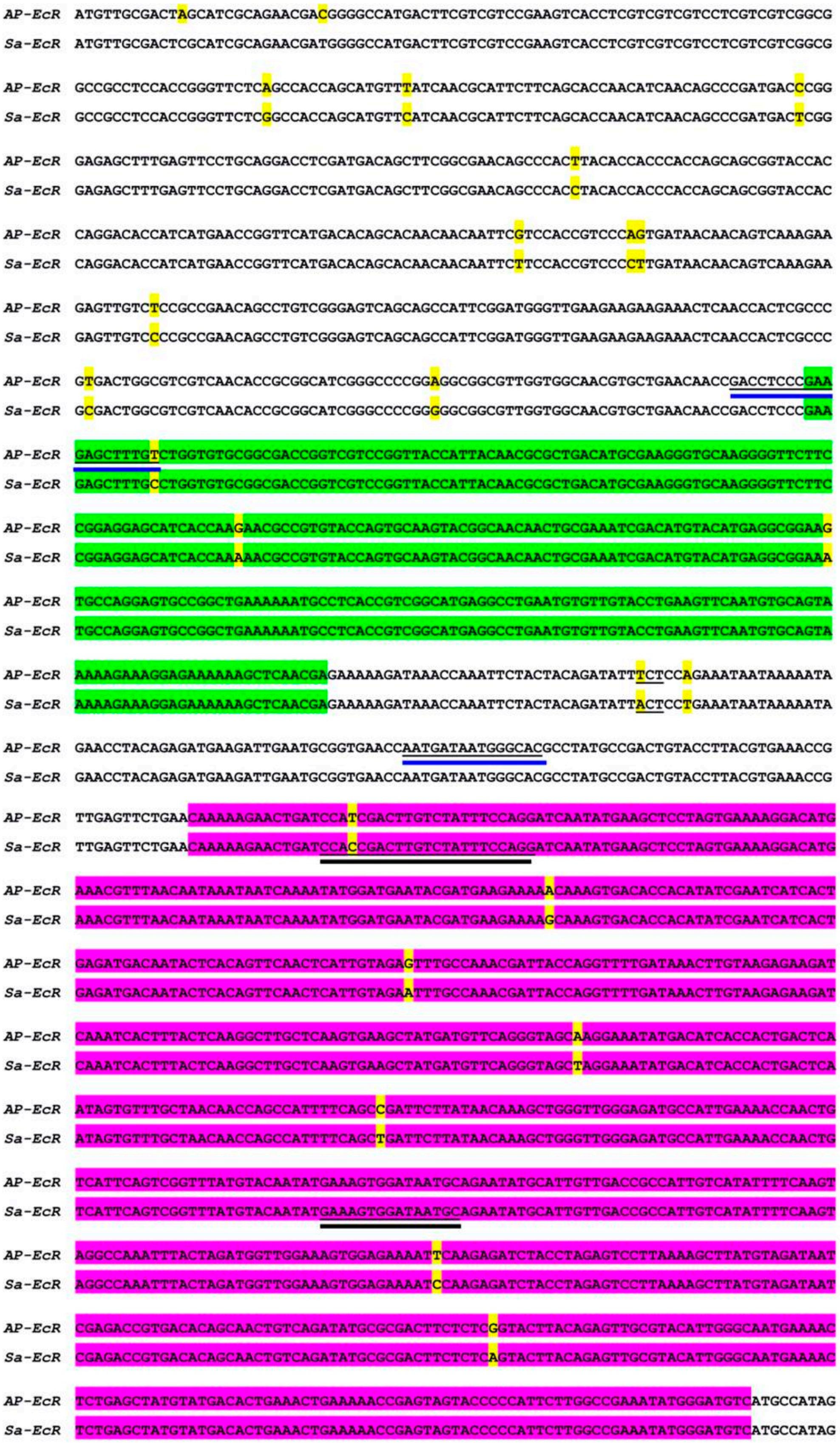

(**a**)

Figure S1. *Cont.*

```
AP-USP ATGTTCAAGAAAGAAAAACCCATGATGTCCGTGTC****GGCCATCATACAGAGCA**GGGCGGCGCATCACCACTGGGGC
Sa-USP ATGCCACTCAGCGAGCTGTCACTGTCGCCCCTGTCATTCGGCTACCGCAATCTCCATTGAACGACCATGGACGGCACCGAA

AP-USP AGAGGATTAAGATTGGACAATAATATGTCACTGGGTTCAATGGGTCCTCAGTCACCTCTAGACCTCAAACCCGACACGGCA
Sa-USP CGAGGTTTAAGATTGGACAATAATATGTCACTGGGTTCAATGGGTCCTCAGTCACCTCTAGACCTCAAACCCGACACAGCA

AP-USP ACTCTAATGGTCAATTTCAGCCCTCCGGGAGCTCCTCTAAGTCCTGCAGGATTATACAGTGTCGACCGGAACAATATGATG
Sa-USP ACTCTAATGGTTAATTTCAGCCCTCCGGGAGCTCCTCTAAGTCCTGCAGGATTATACAGCGTCGACCGGAACAGTATGATG

AP-USP AATAATTCTTGCAACGTACAAGACTCTCCGAATTACCCGCCCAACCATCCGCTCAGCGGTTCGAAACATCTGTGCTCCATA
Sa-USP AATAATTCTTGCAATGTACAAGACTCTCCGAATTACCCGCCCAACCATCCTCTCAGCGGTTCGAAGCATCTGTGCTCCATA

AP-USP TGCGGCGATCGCGCCAGCGGAAAACATTACGGAGTGTACAGCTGCGAGGGGTGCAAAGGGTTCTTCAAACGTACCGTGAGG
Sa-USP TGCGGCGATCGCGCCAGTGGAAAACATTACGGAGTGTACAGCTGCGAGGGGTGCAAAGGGTTCTTTAAACGCACCGTGAGG

AP-USP AAGAATTTGTCTTATGCGTGTCGCGAAGAAAACAAATGCATCATCGACAAGCGCCAACGAAATCGGTGCCAGTACTGCAGG
Sa-USP AAGAATTTGTCTTATGCGTGTCGCGAAGAAAACAAATGCATCATCGACAAGCGCCAACGAAATCGGTGTCAGTACTGCAGG

AP-USP TATCAAAAAATGTTTGACCATGGGCATGAAAAGAGAAGCCGTGCAGGAGGAAAGGCAACGTACAAAAGAACGAGATCATAAT
Sa-USP TATCAAAAAATGTTTGACCATGGGCATGAAAAGAGAAGCCGTGCAGGAGGAAAGGCAACGTACAAAAGAACGAGATCATAAT

AP-USP AGCATAGAAGTTGAACCCACGAGCAGTTCTAATACTGATATGCCAGTGGAACTTATATTAAGGGCTGAGAATAAAGCTGAT
Sa-USP AACATAGAAGTTGAACCCACGAGCAGTTCTAATACTGATATGCCAGTGGAACTTATATTAAGGGCTGAGAATAAAGCTGAT

AP-USP GCTATAAAGACTGAACAACAGTATATAGAGCAACAACATCCTCAACATACTGTTGGTGCTATTTGTCAAGCAACTGACAAG
Sa-USP GCTATAAAGACTGAACAACAGTATATAGAGCAACAACATCCTCAACATACTGTTGGTGCTATTTGTCAAGCAACTGACAAG

AP-USP CAGTTAATACAACTTGTTGAGTGGGCCAAGCATATACCGCATTTTAAAAATTTACCTCTAGGCGATCAAGTTTTGTTATTG
Sa-USP CAGCTAATACAACTTGTTGAGTGGGCCAAGCATATACCGCATTTTAAAAATTTACCTCTAGGCGATCAAGTTTTGTTATTG

AP-USP AGAGCTGGTTGGAATGAGTTGATGATTGCAGCATTTTCCCATAGATCAATCAGTGTAAAGGATGGTATAGTCTTAGCTACT
Sa-USP AGAGCTGGTTGGAATGAGTTGATGATTGCAGCATTTTCCCATAGATCAATCAGTGTAAAAGATGGTATAGTCTTAGCTACT

AP-USP GGACTTACTGTTGACAGAGATTCAGCTCACCAAGCTGGTGTTGAAGCTATATTTGATCGTGTACTCACTGAACTCGTTGCT
Sa-USP GGACTTACTGTTGACAGAGATTCAGCTCACCAAGCTGGTGTTGAAGCTATATTTGATCGTGTACTCACTGAACTCGTTGCT

AP-USP AAAATGAGAGATATGGGTATGGATAGAACAGAGCTTGGCTGTTTACGTACTATTATTCTTTTTAATCCAGGTTCAAAAGGT
Sa-USP AAAATGAGAGATATGGGTATGGATAGAACAGAACTTGGCTGTTTACGTACTATTATTCTTTTTAATCCAGGTTCAAAAGGT

AP-USP TTGCAGTCTGTGAATGAAGTGGAAGTACTACGTGATAAGGTTTATGTTGCGTTAGAAGAATATTGTCGTACAACACATCCA
Sa-USP TTGCAGTCTGTGAATGAAGTGGAAGTACTACGTGATAAGGTTTATGTTGCGTTAGAAGAATATTGTCGTACAACACATCCA

AP-USP GAAGAACCTGGGCGATTTGCTAAACTACTTCTTCGGCTTCCTTCATTACGTTCAATTGGTTTAAAATGTCTGGAACATTTA
Sa-USP GAAGAACCTGGGCGATTTGCTAAACTACTTCTTCGGCTTCCTTCATTACGTTCAATTGGTTTAAAATGTCTGGAACATTTA

AP-USP TTCTTTTATAAACTTATTGGAGATTCACCAATCGATACATTTTTAATGGAAGTTCTTGAATCATCGTCACATGACGTTCAA
Sa-USP TTCTTTTATAAACTTATTGGAGATTCACCAATTGATACATTTTTAATGGAAGTTCTTTAATCATCGTCACATGACGTTCAA

AP-USP GTAGCTACATGA
Sa-USP GTAGCTACATGA
```

(**b**)

Figure S1. *Cont.*

```
AP-EcR  MLRLASQNDGAMTSSSEVTSSSSSSSAAASTGFSATSMFINAFFSTNINSPMTRESFEFLQDLDDSFGEQPTYTTHQQRYH
Sa-ECR  MLRLASQNDGAMTSSSEVTSSSSSSSAAASTGFSATSMFINAFFSTNINSPMTRESFEFLQDLDDSFGEQPTYTTHQQRYH

AP-EcR  QDTIMNRFMTQHNNNSSTVPVITTVKEELSPPNSLSGVSSHSDGLKKKKLNHSPVTGVVNTAASGPGGGVGGNVLNNRPPE
Sa-ECR  QDTIMNRFMTQHNNNSSTVPLITTVKEELSPPNSLSGVSSHSDGLKKKKLNHSPATGVVNTAASGPGGGVGGNVLNNRPPE

AP-EcR  ELCLVCGDRSSGYHYNALTCEGCKGFFRRSITKNAVYQCKYGNNCEIDMYMRRKCQECRLKKCLTVGMRPECVVPEVQCAV
Sa-ECR  ELCLVCGDRSSGYHYNALTCEGCKGFFRRSITKNAVYQCKYGNNCEIDMYMRRKCQECRLKKCLTVGMRPECVVPEVQCAV

AP-EcR  KRKEKKAQREKDKPNSTTDISPEIIKIEPTEMKIECGEPMIMGTPMPTVPYVKPLSSEQKELIHRLVYFQDQYEAPSEKDM
Sa-ECR  KRKEKKAQREKDKPNSTTDITPEIIKIEPTEMKIECGEPMIMGTPMPTVPYVKPLSSEQKELIHRLVYFQDQYEAPSEKDM

AP-EcR  KRLTINNQNMDEYDEEKQSDTTYRIITEMTILTVQLIVEFAKRLPGFDKLVREDQITLLKACSSEAMMFRVARKYDITTDS
Sa-ECR  KRLTINNQNMDEYDEEKQSDTTYRIITEMTILTVQLIVEFAKRLPGFDKLVREDQITLLKACSSEAMMFRVARKYDITTDS

AP-EcR  IVFANNQPFSADSYNKAGLGDAIENQLSFSRFMYNMKVDNAEYALLTAIVIFSSRPNLLDGWKVEKIQEIYLESLKAYVDN
Sa-ECR  IVFANNQPFSADSYNKAGLGDAIENQLSFSRFMYNMKVDNAEYALLTAIVIFSSRPNLLDGWKVEKIQEIYLESLKAYVDN

AP-EcR  RDRDTATVRYARLLSVLTELRTLGNENSELCMTLKLKNRVVPPFLAEIWDVMP*
Sa-ECR  RDRDTATVRYARLLSVLTELRTLGNENSELCMTLKLKNRVVPPFLAEIWDVMP*
```

(**c**)

```
AP-USP *MFKKEKPMMSVSAIIQSRAAHHHWGRGLRLDNNMSLGSMGPQSPLDLKPDTATLMVNFSPPGAPLSPAGLYSVDRNNMMN
Sa-USP MPLSELSLSPLSFGYRNLHTTMDGTERGLRLDNNMSLGSMGPQSPLDLKPDTATLMVNFSPPGAPLSPAGLYSVDRNSMMN

AP-USP NSCNVQDSPNYPPNHPLSGSKHLCSICGDRASGKHYGVYSCEGCKGFFKRTVRKNLSYACREENKCIIDKRQRNRCQYCRY
Sa-USP NSCNVQDSPNYPPNHPLSGSKHLCSICGDRASGKHYGVYSCEGCKGFFKRTVRKNLSYACREENKCIIDKRQRNRCQYCRY

AP-USP QKCLTMGMKREAVQEERQRTKERDHNSIEVEPTSSSNTDMPVELILRAENKADAIKTEQQYIEQQHPQHTVGAICQATDKQ
Sa-USP QKCLTMGMKREAVQEERQRTKERDHNNIEVEPTSSSNTDMPVELILRAENKADAIKTEQQYIEQQHPQHTVGAICQATDKQ

AP-USP LIQLVEWAKHIPHFKNLPLGDQVLLLRAGWNELMIAAFSHRSISVKDGIVLATGLTVDRDSAHQAGVEAIFDRVLTELVAK
Sa-USP LIQLVEWAKHIPHFKNLPLGDQVLLLRAGWNELMIAAFSHRSISVKDGIVLATGLTVDRDSAHQAGVEAIFDRVLTELVAK

AP-USP MRDMGMDRTELGCLRTIILFNPGSKGLQSVNEVEVLRDKVYVALEEYCRTTHPEEPGRFAKLLLRLPSLRSIGLKCLEHLF
Sa-USP MRDMGMDRTELGCLRTIILFNPGSKGLQSVNEVEVLRDKVYVALEEYCRTTHPEEPGRFAKLLLRLPSLRSIGLKCLEHLF

AP-USP FYKLIGDSPIDTFLMEVLESSSHDVQVAT*
Sa-USP FYKLIGDSPIDTFLMEVL*SSSHDVQVAT*
```

(**d**)

Figure S1. The alignments of the coding sequences and the deduced amino acid sequences of *EcR* and *USP* orthologs in grain aphid and pea aphid. (**a**) Alignment of the coding sequences of *EcR* between grain aphid and pea aphid; (**b**) Alignment of the coding sequences of *USP* between grain aphid and pea aphid; (**c**) Alignment of the amino acid sequences of EcR between grain aphid and pea aphid; (**d**) Alignment of the amino acid sequences of USP between grain aphid and pea aphid. The sequences shadowed with green color indicate C domain of *EcR* and *USP*, whereas these shadowed with pink color indicate E domain of *EcR* and *USP*. Differences between grain aphid and pea aphid are highlighted in yellow. The short sequences underlined are primer pairs. Primer pairs for amplification of the fragment for dsRNA synthesis in pea aphid are marked with blue lines, while these in grain aphid are marked with black lines. AP-EcR, Sa-EcR and AP-USP, AP-USP, represent the EcR and USP orthologs from pea aphid and grain aphid, respectively.
